# Childhood infection and adult schizophrenia: A meta-analysis of population-based studies

**DOI:** 10.1016/j.schres.2012.05.023

**Published:** 2012-08

**Authors:** Golam M. Khandaker, Jorge Zimbron, Christina Dalman, Glyn Lewis, Peter B. Jones

**Affiliations:** a Department of Psychiatry, University of Cambridge, Cambridge, UK; b Cambridgeshire and Peterborough NHS Foundation Trust (CPFT), Cambridge, UK; c Division of Public Health Epidemiology, Department of Public Health Sciences, Karolinska Institutet, Stockholm, Sweden; d Academic Unit of Psychiatry, School of Social and Community Medicine, University of Bristol, Bristol, UK

**Keywords:** CNS, Central Nervous System, ICD, International Classification of Diseases, DSM, Diagnostic and Statistical Manual of Mental Disorders, 95% CI, 95% Confidence Interval, Adult, Schizophrenia, Psychotic Disorders, Neurodevelopment, Childhood, Infection, Inflammation, Central Nervous System, Meningitis, Illness, Meta-analysis

## Abstract

**Objective:**

To determine whether exposures to infectious illness during childhood involving the CNS or elsewhere is associated with adult schizophrenia or other psychoses.

**Method:**

Systematic review and meta-analysis of published literature identified by electronic and manual search meeting three inclusion criteria: population-base, objective assessment of childhood infection at the individual level, standard definition of adult psychotic outcomes. We calculated risk ratio for all CNS infection, and separately for viral and bacterial infection in relation to non-affective psychosis and schizophrenia, which was combined in meta-analysis.

**Results:**

Seven studies were included. Meta-analysis involving 2424 cases and over 1.2 million controls showed CNS viral infection was associated with nearly two-fold increased risk of adult non-affective psychosis (risk ratio 1.70; 95% CI 1.13–2.55; *p* = 0.01). There was no significant heterogeneity between studies (*p* = 0.26; *I*^*2*^ = 20%). Separate meta-analysis involving 1035 cases and over 1.2 million controls suggested all childhood CNS infections, particularly viral infections, may be associated with nearly two-fold risk of adult schizophrenia. However, there was evidence of some heterogeneity between these studies (*p* = 0.07; *I*^*2*^ = 70%). CNS bacterial infections were not associated with risk of psychosis. Data on childhood infections with no obvious involvement of the CNS is insufficient.

**Conclusions:**

These findings indicate childhood CNS viral infections increase the risk of adult psychotic illness. Possible mechanisms may include both direct effects of pathogens, and the effects of inflammatory response on the developing brain.

## 1 Introduction

Interference with brain development from early life infections is in line with a neurodevelopmental view of schizophrenia where abnormalities in early brain development contribute to the causes of the disorder (Murray and Lewis, 1987; Weinberger, 1987). Empirical evidence for the relevance of abnormal neurodevelopment comes from birth cohort studies demonstrating delays in motor milestone, and deficits in premorbid cognitive function in future cases of schizophrenia (Jones et al., 1994; Cannon et al., 2000; Cannon et al., 2002a; Khandaker et al., 2011a). So far, studies have linked exposure to a wide range of prenatal maternal infections (and inflammatory response) to risk of schizophrenia in adult offspring (Brown and Derkits, 2010; Khandaker et al., 2012).

The human brain continues to develop throughout childhood and well into early adulthood (de Graaf-Peters and Hadders-Algra, 2006). Several studies have investigated the effects of infectious illness, particularly that of the central nervous system (CNS), in early years of life, in relation to adult schizophrenia (Rantakallio et al., 1997; Leask et al., 2002; Suvisaari et al., 2003; Koponen et al., 2004; Abrahao et al., 2005; Dalman et al., 2008; Weiser et al., 2010). However, findings from these studies are somewhat contradictory (Abrahao et al., 2005; Weiser et al., 2010). Fundamental questions yet to be settled include whether an association between childhood CNS infection and adult schizophrenia really exists, whether this is confined to a particular type of infection (such as viral, bacterial or specific infectious agent) (Weiser et al., 2010), and the roles of infection, inflammation and immunity in the pathogenesis of schizophrenia (DeLisi, 1996). The effects of common childhood infections not directly affecting the CNS are also unclear (Leask et al., 2002). As for prenatal infections, associations between a range of childhood infections and adult schizophrenia would suggest that a common mechanism, possibly involving inflammatory response and the immune system, may be important for pathogenesis of schizophrenia. Alternatively, associations with a particular infectious agent (or group) might suggest more specific mechanisms.

We report a systematic review and meta-analyses of population-based studies concerning early life CNS infection and risk of adult schizophrenia and other non-affective psychosis. We summarise the risk of psychotic illness associated with exposure to CNS infections, overall, to viral/bacterial infections and to specific infectious agents. We discuss associations between common childhood non-CNS infections and adult psychosis, and the effects of early life CNS infections on neurocognitive development.

## 2 Methods

### 2.1 Search strategy

We searched Medline-PubMed and Embase databases from their respective inceptions to February 2012 for studies based on human samples and published in the English language. Search terms included both indexing terms (MeSH or Key words) and free texts: [(infection OR inflammation OR illness OR Central Nervous System OR CNS OR viral OR bacterial OR meningitis) AND (childhood OR early life OR neonatal) AND (schizophrenia OR psychotic disorder)]. We also hand searched reference lists of included studies, and wrote to prominent authors for unpublished data.

### 2.2 Study selection

Included studies used (i) population-based cohort or case–control designs, (ii) clinical examination and/or serology to diagnose infection at the individual level from birth until age 18 years, and (iii) contemporary ICD or DSM to define outcome of schizophrenia and other psychotic disorder. Studies that used population level data, such as prevalence of influenza, rather than direct measurement of infection at the individual level were excluded. Studies based on neonatal blood samples that measured specific antibodies, such as IgG to *Toxoplasma gondii*, were also excluded; such antibodies represent fetal exposure to maternal infection rather than newly acquired infection after birth.

### 2.3 Data extraction

Electronic search and study selection was carried out by two researchers working independently (GMK and JZ). They examined all titles and abstracts, obtained full texts of potentially relevant papers and applied inclusion criteria. All studies meeting inclusion criteria were critically appraised using the Newcastle–Ottawa Scale (NOS) for assessing the quality of non-randomised studies in meta-analyses (Wells et al., 2000). On this scale case–control and cohort studies can earn a maximum score of 9 based on sample selection, comparability and exposure criteria. Duplicate reports were excluded (Gattaz et al., 2004).

### 2.4 Data synthesis

Meta-analysis was restricted to CNS infections as most studies included exposure to only this type of infection. Following quality assessment, we decided to combine results from three population-based cohorts (Swedish, 1966 Finnish and 1958 British) in meta-analyses (Leask et al., 2002; Koponen et al., 2004; Dalman et al., 2008). Out of two available reports from the Finnish cohort the one with longer follow-up (Koponen) was included in meta-analysis (Rantakallio et al., 1997; Koponen et al., 2004). Remaining studies were excluded because they either did not include a control group (Suvisaari et al., 2003), or had methodological weaknesses regarding sample selection which might introduce bias (Abrahao et al., 2005; Weiser et al., 2010) (described below).

For meta-analysis, from each study we calculated cumulative incidence ratio (or risk ratio) of both non-affective psychosis and schizophrenia for exposure to any CNS infection, further into viral and bacterial infection. We used both fixed and random effect models for the calculation of pooled effect estimates. Heterogeneity between study samples was assessed using Cochrane's heterogeneity statistic *Q* (Egger et al., 2001). The *I*^2^ statistic was calculated to express the fraction of variation between studies that was due to heterogeneity (Higgins et al., 2003). We also carried out sensitivity analyses to examine effects of individual studies on effect estimates. Data were analysed using Comprehensive Meta-Analysis Version 2.0 (Biostat Inc, NJ, USA).

## 3 Results

**Fig. 1:**
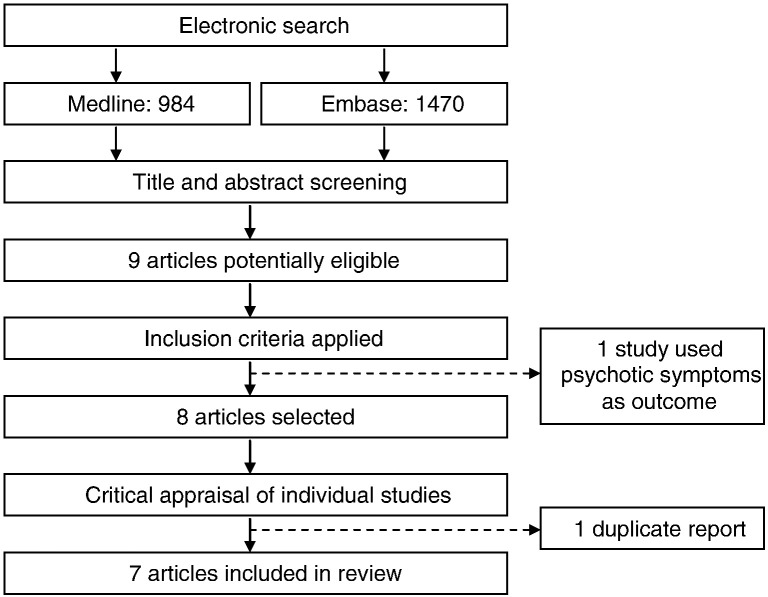
Selection of studies for the review of early life infection and schizophrenia.

We identified 7 population-based studies of early life infection and adult schizophrenia and other psychotic disorders (Fig. 1). Individual study characteristics including their strengths and limitations are presented in Table 1.

### 3.1 Study population and control selection in studies included in review

Four studies from three population-based cohorts compared risk of schizophrenia and other psychoses between those with a history of childhood infection and the rest of the cohort (Rantakallio et al., 1997; Leask et al., 2002; Koponen et al., 2004; Dalman et al., 2008). One was a Swedish national cohort of nearly 1.2 million individuals born between 1973 and 1985 (Dalman et al.,2008). Two reports from the Finnish birth cohort differed only in length of follow-up (Table 1); both were based on the same 11,017 individuals due to be born in Northern Finland in 1966 who were alive and living in Finland at age 16 years (91% of original cohort) (Rantakallio et al., 1997; Koponen et al., 2004). Another study was based on 14,501 individuals from the 1958 British birth cohort with information on childhood infections by age 11 years (83% of original cohort) (Leask et al., 2002). Results from these cohorts were included in meta-analyses.

One study was based on individuals who were admitted to a hospital in Sao Paolo, Brazil with meningococcal meningitis during an epidemic between 1971 and 1974 (Abrahao et al., 2005). Controls were unexposed siblings who were closest in age to cases. However, this study suffered high attrition (96%). Weiser et al. searched records of seven hospitals that cover nearly half of the population of Israel for cases who were hospitalised during childhood with a CNS infection (Weiser et al., 2010). Unlike previous studies, controls were children admitted to hospitals for acute gastroenteritis. The use of a control group who were also suffering from an infection may have introduced bias (see Discussion section). Finally, the seventh study reported incidence of schizophrenia among 320 individuals with laboratory confirmed CNS viral infection during childhood, but did not include a control group (Suvisaari et al., 2003). These studies were excluded from meta-analyses due to methodological issues discussed above.

### 3.2 Meta-analysis of childhood CNS infection and risk of later non-affective psychoses including schizophrenia

**Fig. 2:**
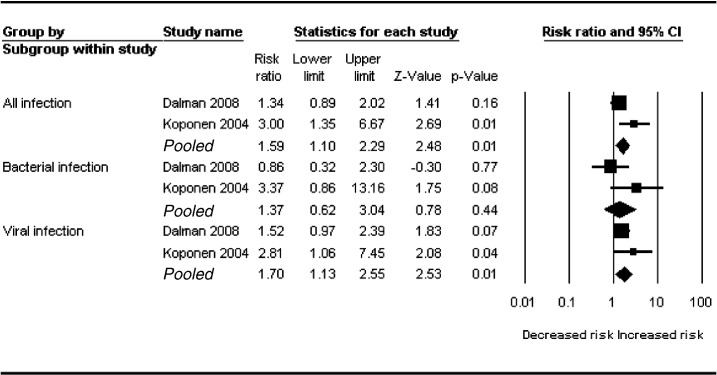
Meta-analysis of childhood CNS infection and adult non-affective psychosis including schizophrenia.

Data was available from two cohorts (Dalman, Koponen); totalling 2424 cases and over 1.2 million controls (Fig. 2). Using a fixed effect model, only viral infection was found to be associated with a significant increase in risk, risk ratio 1.70 (95% CI 1.13–2.55; *p* = 0.01). There was no evidence of significant heterogeneity between studies (*p* = 0.26; *I*^*2*^ = 20%). There was no statistically significant association between bacterial infection and adult psychosis. All results were virtually unchanged using a random effect model.

### 3.3 Meta-analysis of childhood CNS infection and risk of later schizophrenia

**Fig. 3:**
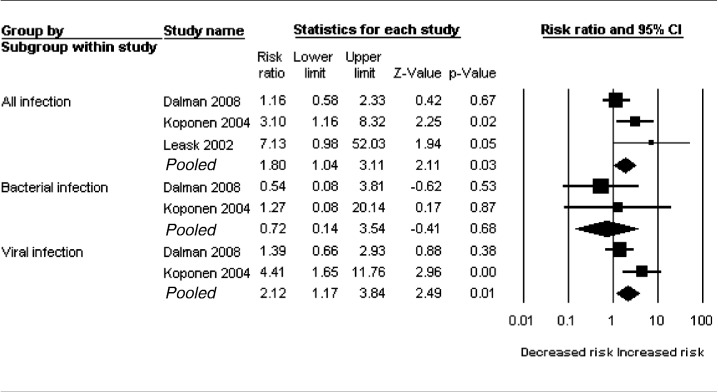
Meta-analysis of childhood CNS infection and adult schizophrenia.

Data were available from all three eligible cohorts (Dalman, Koponen and Leask) for this analysis, totalling 1035 cases and over 1.2 million controls (Fig. 3). However, data from the 1958 British birth cohort was not available separately for viral and bacterial infections, and therefore, was included only in the analysis of all infections. Using a fixed effect model, nearly two-fold increased risk of adult schizophrenia was observed for all childhood CNS infections (risk ratio 1.80; 95% CI 1.04–3.11; *p* = 0.03). However, there was evidence of some heterogeneity between these studies (*p* = 0.10; *I*^*2*^ = 55%). Sensitivity analysis excluding Leask et al. did not change results significantly. Similar to non-affective psychosis, there was no significant association between bacterial CNS infection and adult schizophrenia. However, over two-fold increased risk of adult schizophrenia was observed for CNS viral infections (risk ratio 2.12; 95% CI 1.17–3.84; *p* = 0.01). There was evidence of some heterogeneity between these studies (*p* = 0.07; *I*^*2*^ = 70%).

In a separate analysis, all effect estimates fell short of statistical significance when a random effect model was applied. Risk ratios for all and viral CNS infections were 2.26 (95% CI 0.88–5.84; *p* = 0.09) and 2.37 (95% CI 0.77–7.28; *p* = 0.13), respectively.

### 3.4 Specific infectious agents and schizophrenia and other psychotic disorders

Only one study (Dalman et al.,2008) had the statistical power to adequately examine risk associated with specific infectious agents (Table 2). In this study half of all viral infections were caused by enterovirus, followed by mumps (18%) and varicella zoster (5%). At follow-up, two out of 63 children (3%) with CNS cytomegalovirus infection, and six out of 1162 (0.5%) with CNS mumps virus infection developed non-affective psychosis (Dalman et al.,2008). None of the other viral or bacterial infections was associated with significant risk of psychosis.

Coxsackie B5 virus (CBV-5) (an enterovirus) meningitis in childhood also seemed to be associated with high incidence of adult schizophrenia. Between July and August 1966 there was an epidemic of CBV-5 meningitis which affected 17 newborns in Finland (Rantakallio et al., 1970). Sixteen of these individuals were included in the 1966 Finnish birth cohort, of which two developed schizophrenia by age 28 years (Rantakallio et al., 1997).

Only one study examined effects of common childhood infections with no obvious involvement of the CNS (measles, mumps, varicella, German measles, whooping cough, scarlet fever, infectious hepatitis and rheumatic fever) as well as meningitis and tuberculosis (Leask et al., 2002). However, none of the eight common childhood infections were reported to be associated with increased risk of schizophrenia or other psychosis.

### 3.5 Characteristics of exposed individuals who developed psychotic illness

Two general population cohorts reported detail characteristics of individuals with adult schizophrenia or other psychosis who were exposed to a CNS infection during childhood (Rantakallio et al., 1997; Dalman et al., 2008). All of them suffered from a viral or bacterial infection. In the Swedish cohort, out of 23 exposed cases 14 (60%) were male, and 8 (35%) had a diagnosis of schizophrenia (Dalman et al.,2008). Age at the time of infection was evenly distributed throughout childhood (from 0 to 12.4 years) among exposed cases. Six (26%) of these individuals had a history of substance misuse (most commonly cannabis) before the onset of psychotic illness (Dalman et al.,2008).

In the 1966 Finnish birth cohort six cases were exposed (Rantakallio et al., 1997). All four cases of schizophrenia suffered from a viral infection (two suffered from CBV-5 meningitis), while both cases of other psychosis suffered from bacterial meningitis. Age at the time of infection among exposed cases ranged from 9 days to 7.2 years.

### 3.6 Neurocognitive development and childhood infection

Two birth cohorts indicate risk of adult psychotic illness following early life CNS infection may not be directly related to childhood neurological abnormalities (Rantakallio et al., 1997; Leask et al., 2002). In the 1958 British birth cohort both adult schizophrenia and affective psychosis was associated with increased neurological soft signs at ages 7 and 11 years (Leask et al., 2002). Infectious illness and neurological soft sings were also correlated in the population as a whole. As not all infection was associated with schizophrenia, this is, therefore, unlikely that high neurological soft sign in future cases arose solely as a result of an infection.

The 1966 Finnish birth cohort also reported more neurological abnormalities in the psychosis group; however, they were not related to childhood CNS infection (Rantakallio et al., 1997). For example, school performance at age 16 years was significantly poorer in those who developed a psychotic illness later in life than controls, but cases with CNS infection did not do any worse than unexposed cases.

## 4 Discussion

Our meta-analysis indicates an association between childhood CNS viral infection and risk of adult non-affective psychosis. There is also some evidence that all childhood CNS infections, particularly viral infections, may be associated with increased risk of adult schizophrenia. These findings suggest that the vulnerable period during which harmful events with respect to brain development may increase risk of psychotic illness is not confined to prenatal period (Mednick et al., 1988; Brown and Derkits, 2010; Khandaker et al., 2012). Studies included in our meta-analyses were large, and methodologically robust. They compared risk of adult psychosis between those who were exposed to childhood CNS infection with the rest of the cohort, and defined exposure and outcome using standard methods. Based on representative, general population datasets the evidence is of high quality, with good internal and external validity. However, we acknowledge that our results are based on only two/ three cohorts as several studies were excluded due to methodological reasons. Thus, we could not carry our formal tests for publication bias; this cannot be ruled out and additional studies are required.

We reviewed seven population-based studies, and all except one (Weiser) reported increased risk of adult psychosis for childhood CNS infection. One explanation for discrepant finding from this study may be bias arising from choice of controls. Unlike other studies, controls in this study also suffered from an infection (acute gastroenteritis) serious enough to warrant hospital admission (Weiser et al., 2010). Interference with brain development in some controls is possible via immunological mechanisms (discussed below).

Besides, in some controls gastroenteritis may have been caused by undiagnosed neurotropic agents linked with the risk of psychosis. For example, Coxsackie B5 virus (CBV-5) is highly neurotropic but can also present with symptoms of acute gastroenteritis (Pelon et al., 1996). Evidence suggests childhood CBV-5 infection may be related to schizophrenia. For example, cumulative incidence of schizophrenia among 16 individuals with childhood CBV-5 meningitis was 12.5% in the 1966 Finnish birth cohort (Rantakallio et al., 1997). Possibility of misclassification of exposure in the Israeli sample also cannot be ruled out as a specific virus/bacteria was not identified in over half of the exposed children (Weiser et al., 2010).

In the Swedish cohort the cumulative incidence of non-affective psychosis and schizophrenia was 0.19% and 0.07%, respectively, much lower than the other studies included in this review. Published reports suggest that incidence of schizophrenia and other non-affective psychosis may be around 20/100 k/year (Eaton et al., 2011; Kirkbride et al., 2012). The restriction to hospitalised cases of psychosis and relatively short duration of follow up may explain low incidence of psychosis in this sample. The latter is in common with most of the studies included in this review (Table 1). Thus, future studies should allow longer follow-up, and take measures to include schizophrenia patients who are treated in the community (Jorgensen et al., 2010).

The 96% loss to follow-up in the Sao Paolo sample will have almost certainly introduced bias (Abrahao et al., 2005). Overall rates of psychiatric illness were unusually high in this sample (> 60%). The authors suggested that call to a medical review may have attracted a sample in higher need of medical/psychiatric care. This study reported increased risk of schizophrenia for childhood bacterial meningitis, which is in contrast to findings from our meta-analysis. The observed association in this study may be linked to higher virulence of a pandemic meningococcal strain, or to the study methodology.

Only one study that included infections with no obvious involvement of the CNS lacked power to adequately examine associations between individual infections and adult psychosis (Leask et al., 2002). Since recent evidence points towards potentially important effects of peripheral or systemic infections on the brain (discussed below), in future, studies with larger samples are required.

Schizophrenia is associated with abnormalities in early neurodevelopment, such as cognitive and social deficit during the premorbid period (Jones et al., 1994; Cannon et al., 2000, 2002a; Khandaker et al., 2011a). Short and long-term neurocognitive abnormalities including decreased verbal IQ are well documented following early life CNS infections (Christie et al., 2011). Neurological complications have also been reported to accompany systemic infections (Khandaker et al., in press). However, none of the studies reviewed here included any mediation model to properly explore the effects of CNS infection on childhood neurocognitive development and other indicators of schizophrenia. This should be addressed in future investigations.

A range of maternal and obstetric factors have been linked with increased risk of schizophrenia in offspring (Cannon et al., 2002b; Khandaker et al., 2011b). In the 1966 Finnish birth cohort three out of four cases of schizophrenia exposed to viral meningitis also experienced adverse neonatal events (Rantakallio et al., 1997). The authors accounted for perinatal brain damage in the analysis but the possibility of confounding by other factors cannot be ruled out; not least family history of psychosis. Due to lack of individual participant data we were unable to adjust for confounding factors in meta-analyses. However, individual studies adjusted their findings for several confounding factors (Table 1) such that residual confounding is unlikely to be the sole explanation for the observed associations between CNS infection and psychosis.

An association between childhood CNS viral infection and adult non-affective psychosis indicates aetiological mechanisms related to specific infections (or group) may be important. For example, mumps infection was reported to be associated with increased risk of non-affective psychosis in the Swedish cohort. It has been reported that certain genotypes of the mumps virus (type C and D) exhibit greater neurovirulence than others (genotype A) (Tecle et al., 1998). Mumps have been reported to be associated with long-term difficulties with memory and other neurological sequelae in humans (Julkunen et al., 1985).

Maternal infection during pregnancy with *T. gondii*, Rubella, Cytomegalovirus, Herpes Simplex Virus and other microbes (i.e. TORCH infections) have long been known to be associated with mental retardation, cerebral hypoplasia, ventriculomegaly, and other brain and behavioural abnormalities in the offspring (Remington et al., 2006). For example, congenital rubella has been linked with increased risk of schizophrenia, including childhood IQ deficit in exposed cases (Brown et al., 2001). Thus, it is possible childhood infections with specific viral agents may contribute towards increased risk of adult non-affective psychosis by directly interfering with brain development.

However, possible links between all CNS infections and adult schizophrenia suggest mechanisms common to a range of infections (such as inflammatory response) may also have a role. There is evidence of immune dysfunction in patients with schizophrenia as reflected by increased proinflammatory cytokine, such as interleukin 6 (IL-6) (Potvin et al., 2008). This may have links with early life infections. Increased risk of schizophrenia, and structural and functional brain abnormalities relevant to schizophrenia have been reported among individuals exposed to immune activation or infection during fetal life (Brown and Derkits, 2010; Khandaker et al., 2012). Birth cohort studies suggest inflammatory cytokines may mediate the risk of psychosis related to prenatal infection (Buka et al., 2001; Brown et al., 2004).

There is evidence that peripheral or systemic infection can lead to changes in cognition, mood and behaviour (Dantzer et al., 2008). Peripheral inflammatory cytokines, such IL-6 or TNF- α released as result of inflammation can communicate with the brain in a number of ways: via the vagus nerve, active transport, or entry through leaky circumventricular areas in the blood–brain barrier (Dantzer et al., 2008). Once in the brain, the cytokine signal stimulates microglia to secrete cytokines, chemokines, and proteases from its monocyte and macrophage linage. These local inflammatory mediators can affect neuronal function and synaptic plasticity, metabolism and reuptake of neurotransmitters (such as, serotonin, noradrenalin and dopamine), and stimulate the hypothalamic-pituitary-adrenal axis (Dantzer et al., 2008). In healthy volunteers, peripheral immune activation has been shown to increase circulating cytokines, induce anxiety and low mood, and decrease cognitive performance (Reichenberg et al., 2001). It has been suggested cognitive and functional decline following systemic infection in older adults may be linked to the effects of systemic proinflammatory response on the brain (Khandaker and Jones, 2011c).

It has been reported that areas containing previously activated microglia tend to respond more strongly to a new stimulus (Hickie et al., 2009). Thus, childhood infection may have a priming effect on the CNS immune system (Hickie et al., 2009). This is consistent with the ‘two hit’ model of schizophrenia (Bayer et al., 1999), whereby exposure to various risk factors during early life is thought to prime the CNS for a pathologic response to a ‘second hit’ via the same signaling pathway (Maynard et al., 2001).

It has been suggested some genetic vulnerability to schizophrenia may be explained by genetic vulnerability to infection (Stefansson et al., 2009). Recently, a genome wide association study of schizophrenia has reported significant associations between schizophrenia and markers close the major histocompatibility complex region on chromosome six (Stefansson et al., 2009). This region includes several immunity related genes as well as a histone gene cluster relevant to gene expression; also genes involved in brain development, memory and cognition (Stefansson et al., 2009). It is possible that infection, by affecting gene expression or in the presence of pre-existing genetic vulnerabilities, leads to a distinct or pathological immune response. This may, in turn, lead to CNS alterations via cytokines and other mechanisms making these individuals susceptible to developing psychotic illness later in life. Future epidemiological studies should include genetic information as well as inflammatory cytokines and other immunological markers to elucidate effects of early life infection on neurodevelopment, and risk of psychotic illness later in life.

## Role of the funding source

Golam Khandaker is supported by a grant from the Wellcome Trust (Clinical PhD Programme, grant number 094790/Z/10/Z). Peter Jones is also supported by the Wellcome Trust (095844/Z/11/Z & 088869/Z/09/Z) and NIHR (RP-PG-0606-1335). The Wellcome Trust and NIHR had no further role in study design; in the collection, analysis and interpretation of data; in writing of the report; and in the decision to submit the paper for publication.

## Contributors

GMK carried out literature search, meta-analyses and wrote the first draft of the manuscript. JZ carried out literature search and contributed to the manuscript. CD contributed to the manuscript. PBJ and GL provided overall supervision and contributed to the manuscript.

## Declaration of interest

Golam Khandaker, Jorge Zimbron, Christina Dalman and Glyn Lewis report no competing interests. Peter Jones is co-inventor on patent PCT/GB2005/003279 (methods for assessing psychotic disorders). Peter Jones has received research support from GlaxoSmithKline, and directs the National Institute for Health Research Collaborations for Leadership in Applied Health Research and Care for Cambridgeshire and Peterborough (CLAHRC-CP) of which this work forms part.

## Figures and Tables

**Table 1:** Selected population-based studies of childhood infection and schizophrenia and other psychotic disorder.

Study	Design, sample and type of infection	Outcome and diagnostic criteria	Age of infection, max (years)	Data source	Age at follow up (years)	Cumulative incidence of schizophrenia (%)	Adjustment for confounding	NOS scor^a^Study strengths and limitations
Weiser et al. (2010)	Case control:	Schizophrenia, non-affective psychosis and affective disorders by ICD 10	15	Infection: hospital records	Mean: 29.3, SD 6.0	CNS infection = 0.66%	Gender	5
	CNS infection = 3599 (414 bacterial and 3185 viral)			Outcome: last discharge diagnosis from National Psychiatric Hospitalization Case Registry	Range: 20–50 years	CNS Viral = 0.56%		Strength: large sample
	Control = 6371 acute gastroenteritis					CNS Bacterial = 1.45%		Limitations: potential for bias as controls also suffered from an infectious illness, only used hospitalised cases of psychosis, short follow up
						Controls = 0.75%		
Dalman et al. (2008)	Cohort study:	Schizophrenia and non-affective psychosis by ICD 9 and ICD 10	12	Infection and psychosis: Swedish National Inpatient Register	Range: 17–29 years	CNS infection = 0.26%^b^	Age, gender, urbanicity, parental psychosis	7
	CNS infection = 8985 (2435 bacterial and 6550 viral)					CNS Viral = 0.29%^b^		Strengths: large sample, adjusted for important confounders
	Unexposed = 1,178,586					CNS Bacterial = 0.16%^b^		Limitations: only used hospitalised cases of psychosis, short follow up, did not account for attrition and possibility of outcome at the time of exposure assessment.
						Unexposed = 0.19%^b^		
Abrahao et al. (2005)	Case control:	Schizophrenia and other psychosis by ICD 10	4	Infection: hospital records	Mean: 30, SD 5.9	Meningitis = 4.21%	Unaffected siblings served as controls	5
	Meningitis = 190			Outcome: telephone directory search followed by interview with psychiatrist and neurological evaluation		Control = 0%		Strength: reliable assessment of outcome
	Control = 156 unexposed sibling							Limitation: small sample, high attrition and potential for selection bias
Koponen et al. (2004)^c^	Birth cohort:	Schizophrenia by DSM-III-R	14	Infection: hospital dmission and neurological outpatient clinic records	Mean: 31	CNS infection = 2.75%	Social class, gender, perinatal brain damage, mental retardation, childhood epilepsy	7
	CNS infection = 145 (102 viral)			Outcome: Finnish Hospital Discharge Register (FHDR)		CNS Viral = 3.92%		Strengths: large sample
	Unexposed = 10791					CNS Bacterial = 0		Limitations: only used hospitalised cases of psychosis
						Unexposed = 0.88%		
Rantakallio et al. (1997)	Birth cohort:	Schizophrenia, other psychosisand schizophrenia spectrum disorder by DSM-III-R	14	Infection: hospital admission and neurological outpatient clinic records	Mean: 28	CNS infection = 2.75%	Social class, gender, perinatal brain damage, mental retardation, epilepsy and hearing deficits	7
	CNS infection = 145 (102 viral)			Outcome: Finnish Hospital Discharge Register		CNS Bacterial = 0		Strengths: large sample
	Unexposed = 10872					Unexposed = 0.66%		Limitations: only used hospitalised cases of psychosis
Suvisaari et al. (2003)	Cohort study:	Schizophrenia by ICD (8, 9 and 10)	15	Infection: laboratory records	Mean: 32, SD 4.5	All CNS viral = 0.94%	–	3
	CNS viral infections (n = 320)			Outcome: Finnish Hospital Discharge Register	Range: 23–41 years			Strength: reliable assessment of exposure
								Limitation: small sample, no control group used
Leask et al. (2002)	Birth cohort:	Schizophrenia and affective psychosis by Present State Examination (PSE) and CATEGO	11	Infection: examination by school medical officer and interview with mother at age 11 years	Range: 16–28 years	Meningitis:	Gender and social class	6
	Childhood infection = 74^d^			Outcome: National psychiatric hospital admission records		Schizophrenia group = 4.34%		Strength: large sample
	Unexposed = about 12,000					Control group = 0.6%		Limitations: only used hospitalised cases of psychosis, some exposure information relied on maternal report

a New Castle–Ottawa Scale (NOS) score, higher score represents better quality (maximum score 9).

b Non-affective psychosis including schizophrenia.

c Based on 31 year follow of the 1966 Finnish birth cohort previously reported by Rantakallio et al.

d Only study to include non-central nervous system infection in exposure; exposures included measles, chicken pox, mumps, German measles, whooping cough, scarlet fever, infectious hepatitis, rheumatic fever, meningitis and tuberculosis.

**Table 2:** Risk associated with specific infections.

Study	Psychotic disorder	Type of childhood CNS infection	Risk estimate (95% CI)	Measure of risk
Dalman et al. (2008)	Non-affective psychosis^a^	Cytomegalovirus	16.6 (4.30–65.10)	Risk ratio
		Mumps virus	2.7 (1.20–6.20)	
Rantakallio et al. (1997)	Schizophrenia	CBV-5 meningitis	12.5% (− 4.90%–29.90%)	Cumulative incidence
Leask et al. (2002)	Schizophrenia	Tuberculosis^c^	15 (2.00–120.00)	Odds Ratio^b^
	Affective psychosis	Tuberculosis^c^	12 (1.60–91.00)	
		Chicken pox^c^	0.33 (0.20–0.70)	

a Includes schizophrenia.

b Adjusted for social class and gender.

c Not CNS infection.
